# Pancreaticoduodenectomy versus local resection in the treatment of gastrointestinal stromal tumors of the duodenum

**DOI:** 10.1186/1477-7819-11-196

**Published:** 2013-08-14

**Authors:** Bo Zhou, Min Zhang, Jian Wu, Sheng Yan, Jie Zhou, Shusen Zheng

**Affiliations:** 1 Division of Hepatobiliary and Pancreatic Surgery, Department of Surgery, First Affiliated Hospital, School of Medicine, Zhejiang University, Hangzhou 310003, China; 2 Department of Pathology, First Affiliated Hospital, School of Medicine, Zhejiang University, Hangzhou, China

**Keywords:** Gastrointestinal stromal tumor (GIST), Duodenum, Local resection, Pancreaticoduodenectomy, Surgery

## Abstract

**Background:**

Gastrointestinal stromal tumors (GISTs) are the most common mesenchymal neoplasms. However, duodenal GISTs compromise a small and rare subset and few studies have focused on them. We evaluated the surgical management of patients with duodenal GISTs treated by pancreaticoduodenectomy (PD) versus local resection (LR) in our institution and analyzed the postoperative outcomes.

**Methods:**

This was a retrospective review of patients with duodenal GISTs managed in our institution from January 2006 to January 2012. Clinicopathologic findings and disease-free survival (DFS) of duodenal GIST patients were analyzed.

**Results:**

A total of 48 patients were selected. The most common presentation was bleeding (60.4%), and the second portion of the duodenum (35.4%) was the most common dominant site. Of the patients, 34 (70.8%) underwent LR while 14 (29.2%) underwent PD. The surgical margins for all studied patients were free. Patients who ultimately underwent PD were more likely to present with a larger tumor (median size: PD, 6.3 cm vs LR, 4.0 cm; *P* = 0.02) and more commonly presented with a tumor in the second portion of the duodenum (second portion: PD, 64.3% vs LR, 23.5%; *P* = 0.007). The tumors treated by PD had a higher grade of risk compared with LR as defined by National Institutes of Health (NIH) criteria (*P* = 0.019). PD was significantly associated with a longer operation time and a longer hospital stay compared to LR (*P* < 0.001 and *P* = 0.001, respectively). In our study, the median follow-up period was 36 months (range: 0 to 81 months). The 1- and 3-year DFS was 100% and 88%, respectively. From multivariable analysis, the only significant factor associated with a worse DFS was an NIH high risk classification (hazard ratio = 4.24).

**Conclusions:**

The recurrence of duodenal GIST was correlated to tumor biology rather than type of operation. PD was associated with a longer hospital stay and longer operation time. Therefore, LR with clear surgical margins should be considered a reliable and curative option for duodenal GIST and PD should be reserved for lesions not amenable to LR.

## Background

Gastrointestinal stromal tumors (GISTs) are the most common mesenchymal tumors of the gastrointestinal (GI) tract. They can occur in the stomach (45% to 65%), small intestine (15% to 25%), colon and rectum (5% to 10%) or esophagus (5% to 10%) [1]. The clinical manifestations of GISTs are variable and render accurate diagnosis challenging. The current diagnosis of GIST is based on histologic and immunohistochemical criteria, the most important of which is the expression of the receptor tyrosine kinase KIT (CD117, c-Kit) [1,2]. Duodenal GISTs are a very rare presentation, accounting for approximately 20% of tumors in the small intestine and 1% to 4% of all GISTs [3].

Complete surgical resection remains the best option for the treatment of GISTs, although imatinib mesylate, a tyrosine kinase inhibitor, is effective for GISTs. Unlike gastric GISTs, which can be adequately treated by wedge resection instead of formal gastrectomy, the optimal surgical procedures for duodenal GISTs have not been well characterized in the surgical literature [3-9]. Some authors advocate radical procedures like pancreaticoduodenectomy (PD), whereas others support conservative procedures such as segmental duodenectomy and local or wedge resection (LR) based on their biology: since they are encapsulated tumors, GISTs do not widely infiltrate at the microscopic level and rarely metastasize to lymph nodes. In this study, we retrospectively reviewed the clinicopathologic characteristics of duodenal GISTs in our institution and compared the outcomes of patients undergoing PD versus LR with the main objective of determining if LR is a viable treatment option for these tumors.

## Methods

A total of 48 patients who underwent surgical resection for a duodenal GIST were retrospectively reviewed from January 2006 to January 2012 at the First Affiliated Hospital, Zhejiang University School of Medicine. The preoperative diagnosis of a duodenal GIST was made through computed tomography or endoscopy. Furthermore, the precise diagnosis of GIST was made based on the standard histologic criteria. Complete tumor removal, either LR or PD, was the treatment in each case. In general, following LR the duodenal defect was closed primarily when possible or with a Roux-en-Y duodenojejunostomy when primary closure was not possible. Routine lymphadenectomy was not performed. The following characteristics were collected for each patient: age, sex, presenting symptoms, location of primary tumor, pathologic features including tumor size, mitotic count, immunohistochemistry, operative method, complications and the most recent follow-up information. Data on the use of preoperative or postoperative therapies including imatinib and chemotherapy were also recorded.

The primary study end point was disease-free survival (DFS), which was defined as the time from surgery to GIST recurrence. Patients who did not have evidence of local recurrence or metastasis at the last follow-up and those who died from causes unrelated to GIST were excluded from the DFS analysis. Furthermore, we analyzed prognostic factors, including age, sex (male versus female), clinical presentation (asymptomatic versus symptomatic), risk classification (high versus intermediate, low and very low) and operative method (PD versus LR).

### Statistical analysis

Our results are given as medians (plus ranges) and all statistical analyses were performed using the software SPSS 16.0 (SPSS, Chicago, IL) for Windows. Comparisons of the clinicopathologic characteristics between the two surgical groups were assessed using the chi-squared test for dichotomous and categorical variables. DFS was calculated using the Kaplan–Meier method and differences between the groups were evaluated using the log-rank test. Cox proportional hazard models were used to estimate hazard ratios for DFS and to determine independent risk factors. Statistical significance was defined as *P* values less than 0.05, and all tests were 2-sided.

### Consent and statement of ethical approval

Written informed consent was obtained from all participants. This study was approved by the local Ethics Committee at Zhejiang University School of Medicine.

## Results

### Clinicopathologic characteristics of duodenal gastrointestinal stromal tumors

In total, 48 patients who had presented with duodenal GISTs during the study period were included in the analysis (28 men, 20 women). The median age at presentation was 53 years (range: 27 to 89 years). Of 48 duodenal GISTs, 8 (16.7%) were found incidentally during a health examination. The most common presentation of a symptomatic duodenal GIST was gastrointestinal bleeding, which was seen in 29 (60.4%) patients, followed by abdominal discomfort seen in 7 (14.6%), abdominal pain seen in 3 (6.3%) and jaundice seen in 1 (2%; Table 1). None of the patients had a history of neurofibromatosis. The duodenal GISTs were located at the first (D1) (*n* = 11, 22.9%), second (D2) (*n* = 17, 35.4%), third (D3) (*n* = 6, 12.5%) or fourth portion of the duodenum (D4) (*n* = 2, 4.2%), or they involved both D1/D2 (*n* = 8, 16.7%) or D2/D3 (*n* = 4, 8.3%). The median size of the duodenal GISTs was 4.7 cm (range: 2.0 to 15.0 cm). A low mitotic count was found in 75% of the duodenal GISTs. The numbers of patients classified as low risk, intermediate risk and high risk were 28 (58.3%), 11 (22.9%) and 9 (18.8%), respectively. Immunohistochemically, 97.9% of the duodenal GISTs were positive for CD117, 66.7% for CD34, 12.5% for desmin and 10.4% for S-100 (Table 1). Only one GIST was CD117 and desmin negative; however, it stained positively for CD34 and SMA.

**Table 1:** Clinical and pathological characteristics for patients with a duodenal GIST

**Variable**	**All patients (*n *= 48)**
Age	
Median	53 years
Range	27 to 89 years
Gender	
Male	28 (58.3%)
Female	20 (41.7%)
Presentation	
Bleeding	29 (60.4%)
Incidental finding	8 (16.7%)
Abdominal discomfort	7 (14.6%)
Abdominal pain	3 (6.3%)
Jaundice	1 (2%)
Tumor size	
Median	4.7 cm
Range	2.0 to 15.0 cm
Mitotic count	
≤5 mitosis/50 HPF	36 (75.0%)
6 to 10 mitosis/50 HPF	4 (8.3%)
mitosis HPF	8 (16.7%)
NIH risk classification	
Low risk	28 (58.3%)
Intermediate risk	11 (22.9%)
High risk	9 (18.8%)
Site	
D1	11 (22.9%)
D2	17 (35.4%)
D3	6 (12.5%)
D4	2 (4.2%)
D1/D2	8 (16.7%)
D2/D3	4 (8.3%)
CD117	
Positive	47 (97.9%)
Negative	1 (2.1%)
CD34	
Positive	32 (66.7%)
Negative	16 (33.3%)
Desmin	
Positive	6 (12.5%)
Negative	42 (87.5%)
S-100	
Positive	5 (10.4%)
Negative	43 (89.6%)

D1, first part of the duodenum; D2, second part of the duodenum; D3, third part of the duodenum; D4, fourth part of the duodenum; NIH, National Institutes of Health.

### Comparison of clinicopathological features between tumors treated by PD and by LR

All of the patients underwent a curative resection (R0), and there were 14 PDs and 34 LRs. Comparing PD with LR, many of the clinicopathological characteristics in the two cohorts showed no significant differences, including sex, presence of symptoms, complications, Eastern Cooperative Oncology Group (ECOG) scores and recurrence rates (Table 2). However, the age of patients who underwent PD was older (median age: PD, 59 years vs LR, 51 years; *P* = 0.03). Meanwhile, patients who ultimately underwent PD were more likely to present with a larger tumor (median size: PD, 6.3 cm vs LR, 4.0 cm; *P* = 0.02) and more commonly presented with a tumor in the second portion of the duodenum (second portion: PD, 64.3% vs LR, 23.5%; *P* = 0.007). Also, the tumors treated by PD had a higher grade of risk compared with LR as defined by National Institutes of Health (NIH) criteria (*P* = 0.019). In addition, PD was significantly associated with a longer operation time and a longer hospital stay compared to LR (*P* < 0.001 and *P* = 0.001, respectively).

**Table 2:** Comparison between local resection versus pancreaticoduodenectomy for patients with duodenal gastrointestinal stromal tumors

	**Local resection (*n *= 34)**	**Pancreaticoduodenectomy (*n *= 14)**	***P *value**
Male	19 (55.9%)	9 (64.3%)	0.59
Age (years)	51 (27 to 89)	59 (36 to 72)	0.03
Presentation			0.28
Bleeding	23 (64.7%)	6 (42.9%)	
Incidental finding	4 (11.8%)	4 (28.6%)	
Abdominal discomfort	5 (17.6%)	2 (14.3%)	
Abdominal pain	2 (5.9%)	1 (7.1%)	
Jaundice	0	1 (7.1%)	
Size (cm)	4.0 (2.0 to 7.0)	6.3 (2.5 to 15.0)	0.02
Site			
D1	9 (26.5%)	2 (14.3%)	
D2	8 (23.5%)	9 (64.3%)	0.007
D3	6 (17.6)	0	
D4	2 (5.9%)	0	
D1/D2	6 (17.7%)	2 (14.3%)	
D2/D3	3 (8.8%)	1 (7.1%)	
NIH risk criteria			0.019
Low risk	23 (67.7%)	5 (35.7%)	
Intermediate risk	8 (23.5%)	3 (21.4%)	
High risk	3 (8.8%)	6 (42.9%)	
Operation time (mins)	210 (120 to 390)	395 (240 to 600)	<0.001
Postoperative stay (days)	14 (11 to 35)	21 (12 to 46)	0.001
Complications	4 (11.8%)	5 (35.7%)	0.053
ECOG scores	0 (0 to 5)	0 (0 to 1)	0.80
Mortality	2 (5.9%)	0	0.35
Recurrence	1 (2.9%)	2 (14.3%)	0.14

D1, first part of the duodenum; D2, second part of the duodenum; D3, third part of the duodenum; D4, fourth part of the duodenum; ECOG, Eastern Cooperative Oncology Group; NIH, National Institutes of Health.

### Postoperative course details and long-term outcomes

Postoperative complications occurred in nine duodenal GIST patients, comprising five who underwent PD and four who underwent LR. These complications were pancreatic fistulas, duodenal leaks, wound infections, intra-abdominal hemorrhages and death. None of the 48 patients received chemotherapy or preoperative therapy with imatinib, while nine patients (18.8%) received postoperative adjuvant imatinib with no local recurrence or metastasis (Table 3).

**Table 3:** Complications and treatment details after resection of a duodenal gastrointestinal stromal tumor

**Variable**	**Number of patients (percentage of total number of patients)**
Complications	
Pancreatic fistula	6 (12.5%)
Duodenal leak	2 (4.2%)
Intra-abdominal hemorrhage	1 (2.1%)
Wound infection	3 (6.2%)
Death	2 (4.2%)
Gleevec	
Neoadjuvant	0
Adjuvant	9 (18.8%)
Chemotherapy	0
Site of recurrence	
Liver	2 (4.2%)
Local/regional	1 (2.1%)
Treatment of recurrence	
Resection	1 (2.1%)
Other	2 (4.2%)

The median duration of follow-up was 36 (range: 0 to 81) months. During follow-up, of 48 patients, 43 patients were alive and free of recurrence; one patient died due to an intra-abdominal hemorrhage within 1 month of surgery; one patient died 35 months after the operation due to a myocardial infarction; three patients had recurrence and the risk classifications were all high risk: one patient underwent PD because of GIST recurrence after LR (24 months) and two patients who had undergone PD had liver metastases and did not receive further special treatment (18 and 26 months). The 1- and 3-year DFS rates were 100% and 88%, respectively. Multivariable analysis showed that patient age, sex, presence of symptoms and operative method were not associated with DFS. The only significant factor associated with a worse DFS was an NIH high risk classification (hazard ratio (HR) = 4.24, *P* = 0.04) (Table 4).

**Table 4:** Multivariable analysis of factors for being recurrence-free after resection of a duodenal gastrointestinal stromal tumor

**Factor**	**Hazard ratio (95% CI)**	***P *value**
Age (>65 years)	0.52 (0.17 to 1.56)	0.34
Sex (male)	1.52 (0.75 to 3.09)	0.25
Risk (high/low and intermediate)	4.24 (1.06 to 17.05)	0.04
Operative method (PD/LR)	1.65 (0.69 to 3.93)	0.26
Presentation (asymptomatic/symptomatic)	0.92 (0.38 to 2.22)	0.85

*LR*, local resection; *PD*, pancreaticoduodenectomy.

## Discussion

Duodenal GISTs are a rare tumor entity, accounting for 4% of GISTs [10]. The clinical manifestations of duodenal GISTs are variable depending on tumor size and the existence of mucosal ulceration. As in previous reports, in our study, gastrointestinal bleeding was the most common clinical presentation of a duodenal GIST, followed by incidental finding, abdominal discomfort and abdominal pain. In general, duodenal GISTs most frequently involve the second portion of the duodenum, followed by the third portion, fourth portion and first portion. In the current study, we also noted that the second portion of the duodenum (35.4%) was most commonly involved, while we also found a high incidence of lesions in the first portion (22.9%). Furthermore, the median size of duodenal GISTs in our series was 4.7 cm. Similarly, most authors report a smaller size of tumor (4.0 to 5.0 cm) for duodenal GISTs compared with gastric and small bowel GISTs [6,8,11]. Interestingly, we noted that patients who ultimately required PD were more likely to present with a larger tumor and more commonly presented with a tumor in the second portion of the duodenum compared with LR. So we suggest that a tumor in the second portion of the duodenum, and which involves the papilla, pancreas or the duodenal bulb, or if the tumor is large with a high malignant potential, then PD is mandated since this type of tumor usually cannot be locally resected.

Surgical resection with a clear margin is the best option for the treatment of GISTs [7-9,12]. The size of the surgical margin along the segment of the digestive tract is not formally defined; however, there is little submucosal spread in GIST and clear margins of 1 or 2 cm are recommended. Theoretically, the choice of surgical procedure for a duodenal GIST mainly depends on its size, location and proximity to the duodenal papilla. However, the optimal surgical procedure for a duodenal GIST is currently unknown. Various surgical procedures have been reported, such as pancreaticoduodenectomy (PD) and local resection (LR), including wedge or segmental resection. Some authors advocate radical procedures like PD when the tumor is located at the medial wall of the second portion of the duodenum and involves the ampulla of Vater, or if the tumor has involved the adjoining organs [8,9,13]. Whereas, others support LR based on the tumor’s biology; since they are encapsulated tumors, GISTs do not widely infiltrate at the microscopic level and rarely metastasize to lymph nodes. As reported, LR can be used if the resection can achieve histologically clear margins (R0) and can preserve more of the pancreas parenchyma, duodenum and common bile duct without the cost of increased disease recurrence [7,14-16]. LR can be beneficial for patients because it contributes to a better quality of life and does not involve the excessive resection associated with PD. However, the main concern regarding LR is the risk from the margins involved and hence the theoretical increased risk of local recurrence [7].

Whilst there are several case reports and series about the clinicopathological features and frequency of duodenal GISTs, only a few reports compare the oncological long-term outcome of LR versus PD. The largest series of patients with duodenal GISTs (*n* = 156) indicated that prognosis was associated with tumor grade. In this review, 84 patients underwent LR (15 enucleations, 48 segmental resections and 21 wedge resections, 80%) and 21 (20%) underwent PD, and the retrospective analysis did not address the effect of operative method on disease recurrence after the operation [5]. Very recently, Johnston *et al*. retrospectively reviewed 96 patients at five institutions, and there were 58 LRs and 38 PDs. They concluded that factors associated with a worse recurrence-free survival included tumor size, mitotic count, being AJCC stage III disease and an NIH high risk classification rather than surgical approach [9]. Tien *et al*. analyzed nine patients who underwent PD and sixteen who underwent LR. They also showed that type of operation was not correlated to operative risk and disease recurrence [7]. Of thirteen patients reported by Beham *et al.*, eight underwent segmental duodenectomy and five underwent pylorus-preserving PD. They suggested that the operative procedure and type of mutation (the KIT and PDGFRA receptor tyrosine kinase genes and comparative genomic hybridization) did not correlate with long-term survival [17]. In the present study, we noted that LR did not seem to have an adverse impact on the longer-term outcomes of patients. Multivariable analysis showed that operative method was not associated with DFS. The only significant factor associated with a worse DFS was an NIH high risk classification. Furthermore, PD was significantly associated with a longer operation time and a longer hospital stay compared to LR. Collectively, these data indicate that LR might be a reliable and curative option for a significant subset of patients with duodenal GIST, which is in accordance with the report of El-Gendi [18].

In the current study, the 1- and 3-year DFS was 100% and 88%, respectively. This is similar to previous reports, which showed that the DFS rate at 1 to 3 years after a complete surgical resection, for all kinds of resection, varies from 86% to 100% [8,13,16,18]. Prognosis and recurrence are mainly dependent on tumor biology, which is determined by size and mitotic index (Fletcher scale) [19]. In our study, 75% of patients with a duodenal GIST had a low mitotic count, which is consistent with the findings reported by others (72% to 78%) and is lower than for gastric and small bowel tumors (where a mitotic count >5/50 HPF has been found in more than 30% of cases) [6-9,18]. The data from the study by Miettinen *et al*. also indicated that the prognosis for localized duodenal GISTs was poor with a high risk of relapse (>50%) [5]. Due to the earlier presentation, smaller tumors and a lower NIH risk classification, patients with a duodenal GIST may belong in a better prognostic category than other small bowel GISTs.

Imatinib mesylate, a tyrosine kinase inhibitor, plays a key role in the management of GISTs. It can be used in neoadjuvant therapy, adjuvant therapy and to treat tumor recurrence [20]. The role of neoadjuvant imatinib mesylate in the management of GISTs is currently the subject of an ongoing multicenter trial. In our study, 9 of 48 patients received postoperative adjuvant imatinib and there was no local recurrence or metastasis.

## Conclusions

Because the recurrence of a duodenal GIST is correlated to tumor biology rather than type of operation, LR with clear surgical margins is a reliable and curative option for duodenal GISTs, and is compatible with long-term DFS. PD should be reserved for patients where LR is not technically feasible because of the involvement of the papilla of Vater.

## Abbreviations

D1: First part of the duodenum; D2: Second part of the duodenum; D3: Third part of the duodenum; D4: Fourth part of the duodenum; DFS: Disease-free survival; ECOG: Eastern cooperative oncology group; GIST: Gastrointestinal stromal tumor; HR: Hazard ratio; LR: Local resection; NIH: National institutes of health; PD: Pancreaticoduodenectomy

## Competing interests

The authors declare that they have no competing interests.

## Authors’ contributions

BZ analyzed the data and wrote the manuscript. MZ, SY and SSZ commented on and revised the manuscript. MZ, JW, SY, JZ and SSZ built the patient database. All authors read and approved the final manuscript.

## References

[B1] Machairas A Karamitopoulou E Tsapralis D Karatzas T Machairas N Misiakos EP Gastrointestinal stromal tumors (GISTs): an updated experience Dig Dis Sci 2010 11 3315 3327 10.1007/s10620-010-1360-9 20725786

[B2] Dei Tos AP Laurino L Bearzi I Messerini L Farinati F Gastrointestinal stromal tumors: the histology report Dig Liver Dis 2011 11 S304 S309 21459336 10.1016/S1590-8658(11)60586-0

[B3] Winfield RD Hochwald SN Vogel SB Hemming AW Liu C Cance WG Grobmyer SR Presentation and management of gastrointestinal stromal tumors of the duodenum Am Surg 2006 11 719 722 16913316

[B4] Goh BK Chow PK Kesavan S Yap WM Wong WK Outcome after surgical treatment of suspected gastrointestinal stromal tumors involving the duodenum: is limited resection appropriate? J Surg Oncol 2008 11 388 391 10.1002/jso.20954 18163461

[B5] Miettinen M Kopczynski J Makhlouf HR Sarlomo-Rikala M Gyorffy H Burke A Sobin LH Lasota J Gastrointestinal stromal tumors, intramural leiomyomas, and leiomyosarcomas in the duodenum: a clinicopathologic, immunohistochemical, and molecular genetic study of 167 cases Am J Surg Pathol 2003 11 625 641 10.1097/00000478-200305000-00006 12717247

[B6] Yang WL Yu JR Wu YJ Zhu KK Ding W Gao Y Shen QY Lv KZ Zhang Q Yang XJ Duodenal gastrointestinal stromal tumor: clinical, pathologic, immunohistochemical characteristics, and surgical prognosis J Surg Oncol 2009 11 606 610 10.1002/jso.21378 19697360

[B7] Tien YW Lee CY Huang CC Hu RH Lee PH Surgery for gastrointestinal stromal tumors of the duodenum Ann Surg Oncol 2010 11 109 114 10.1245/s10434-009-0761-5 19841981

[B8] Chung JC Chu CW Cho GS Shin EJ Lim CW Kim HC Song OP Management and outcome of gastrointestinal stromal tumors of the duodenum J Gastrointest Surg 2010 11 880 883 10.1007/s11605-010-1170-6 20140534

[B9] Johnston FM Kneuertz PJ Cameron JL Sanford D Fisher S Turley R Groeschl R Hyder O Kooby DA Blazer D 3rd Choti MA Wolfgang CL Gamblin TC Hawkins WG Maithel SK Pawlik TM Presentation and management of gastrointestinal stromal tumors of the duodenum: a multi-institutional analysis Ann Surg Oncol 2012 11 3351 3360 10.1245/s10434-012-2551-8 22878613

[B10] Cubas RF Ballarino EA Nieto FA Diaz MD Local resection of a gastrointestinal stromal tumor of the third portion of the duodenum Am Surg 2012 11 E22 E23 22273294

[B11] Cavallaro G Polistena A D’Ermo G Pedullà G De Toma G Duodenal gastrointestinal stromal tumors: review on clinical and surgical aspects Int J Surg 2012 11 463 465 10.1016/j.ijsu.2012.08.015 22939976

[B12] Blay JY Bonvalot S Casali P Choi H Debiec Richter M Dei Tos AP Emile JF Gronchi A Hogendoorn PC Joensuu H Le Cesne A McClure J Maurel J Nupponen N Ray Coquard I Reichardt P Sciot R Stroobants S Van Glabbeke M Van Oosterom A Demetri GD Consensus meeting for the management of gastrointestinal stromal tumors. report of the GIST consensus conference of 20–21 March 2004, under the auspices of ESMO Ann Oncol 2005 11 566 578 10.1093/annonc/mdi127 15781488

[B13] Machado NO Chopra P Al Haddabi IH Al Qadhi H Large duodenal gastrointestinal stromal tumor presenting with acute bleeding managed by a Whipple resection. a review of surgical options and the prognostic indicators of outcome JOP 2011 11 194 199 21386652

[B14] Sakamoto Y Yamamoto J Takahashi H Kokudo N Yamaguchi T Muto T Makuuchi M Segmental resection of third portion of duodenum for a gastrointestinal stromal tumor: a case report Jpn J Clin Oncol 2003 11 364 366 10.1093/jjco/hyg063 12949065

[B15] Goh BK Chow PK Ong HS Wong WK Gastrointestinal stromal tumor involving the second and third portion of the duodenum: treatment by partial duodenectomy and Roux-en-Y duodenojejunostomy J Surg Oncol 2005 11 273 275 10.1002/jso.20311 16121353

[B16] Buchs NC Bucher P Gervaz P Ostermann S Pugin F Morel P Segmental duodenectomy for gastrointestinal stromal tumor of the duodenum World J Gastroenterol 2010 11 2788 2792 10.3748/wjg.v16.i22.2788 20533599 PMC2883135

[B17] Beham A Schaefer IM Cameron S Von Hammerstein K Füzesi L Ramadori G Ghadimi MB Duodenal GIST: a single center experience Int J Colorectal Dis 2013 11 581 590 10.1007/s00384-012-1432-8 22350270 PMC3639365

[B18] El-Gendi A El-Gendi S El-Gendi M Feasibility and oncological outcomes of limited duodenal resection in patients with primary nonmetastatic duodenal GIST J Gastrointest Surg 2012 11 2197 2202 10.1007/s11605-012-2034-z 23007283

[B19] Fletcher CD Berman JJ Corless C Gorstein F Lasota J Longley BJ Miettinen M O’Leary TJ Remotti H Rubin BP Shmookler B Sobin LH Weiss SW Diagnosis of gastrointestinal stromal tumors: a consensus approach Hum Pathol 2002 11 459 465 10.1053/hupa.2002.123545 12094370

[B20] Cassier PA Blay JY Gastrointestinal stromal tumors of the stomach and duodenum Curr Opin Gastroenterol 2011 11 571 575 10.1097/MOG.0b013e32834bb7f0 21934616

